# The Tyrosine Kinase Inhibitor Sunitinib Affects Ovulation but Not Ovarian Reserve in Mouse: A Preclinical Study

**DOI:** 10.1371/journal.pone.0152872

**Published:** 2016-04-01

**Authors:** Valérie Bernard, Justine Bouilly, Piet Kramer, Nadège Carré, Martin Schlumberger, Jenny A. Visser, Jacques Young, Nadine Binart

**Affiliations:** 1 Inserm U1185, Univ. Paris-Sud, Université Paris Saclay, Le Kremlin-Bicêtre, 94276, France; 2 Department of Internal Medicine, Erasmus MC, Rotterdam, The Netherlands; 3 Institut Gustave Roussy, Département de Médecine nucléaire et Oncologie endocrinienne, Université Paris-Sud, Villejuif, 94800, France; 4 Service d’Endocrinologie et des Maladies de la Reproduction, Assistance Publique des Hôpitaux de Paris, Univ. Paris-Sud, Faculté de Médecine Paris-Sud, Le Kremlin Bicêtre, 94276, France; China Agricultural University, CHINA

## Abstract

The aim of the study was to evaluate ovarian toxicity of tyrosine kinase inhibitor (TKI) sunitinib, since only scarce data are available on gonadal function after this treatment. Six-week-old female mice received orally, once daily, vehicle or sunitinib (50 mg/kg/d) during 5 weeks. Fertility parameters were analyzed from ovulation to litter assessment. Sunitinib exposure significantly reduced (i) corpora lutea number per ovary (1.1 ± 0.38 in sunitinib group versus 4 ± 0.79 in control group, p<0.01) and (ii) serum Anti Müllerian hormone (AMH) levels in sunitinib treated mice (12.01 ± 1.16) compared to control mice (14.33 ± 0.87 ng/ml, p< 0.05). However, primordial and growing follicles numbers per ovary were not different in both groups. After treatment withdrawal, female mice in both groups were able to obtain litters. These data could be helpful to counsel clinicians and patients, when fertility preservation methods are discussed, before TKI treatment in girls and young women.

## Introduction

Last decade, molecular targeted anticancer therapies have been developed. They constitute a major progress, enhancing and rationalizing the therapeutic arsenal in multiple types of cancer in both adult and children. Many of these agents are inhibitors of receptor tyrosine kinases (RTKs) whose overexpression and activity are associated with several types of cancer [1]. Indeed, RTKs activation by their respective ligands induces cell proliferation and survival, inhibits apoptosis and enhances angiogenesis, invasiveness and metastatic potential, notably through MAP-Kinase pathway.

Tyrosine kinases inhibitors (TKIs), as all anticancer drugs, may induce toxicities. Usually TKIs side effects are manageable and include fatigue, diarrhea, nausea, weight loss, hypertension and dermatologic toxicities [2]. Endocrine side effects can also occur, and notably thyroid dysregulation has been reported most in the literature [3]. However, only scarce data are available on gonadal function and most studies address male gonadal toxicity and/or imatinib treatment which was one of the first TKIs to prove antitumoral efficacy. A first study regarding testosterone levels in men treated with imatinib reported abnormal endocrine testicular function in most of them, and 18% of patients developed gynecomastia. Later on, a number of reports confirmed the possible occurrence of gynecomastia after different TKIs uses [4–7]. Another case report presented the occurrence of oligospermia during imatinib treatment [8]. This potential alteration of testicular Sertoli cells function during TKIs treatment has been reported in a recent prospective study [9].

Nevertheless, gonadal TKI treatment tolerance has been poorly studied in women. Only one case of primary ovarian insufficiency was reported [10], then debated [11] in part because ten patients who conceived during imatinib treatment of chronic myeloid leukemia had been described [12]. In fact, the hypothesis of a link between TKIs treatment and primary ovarian insufficiency is plausible, since kinase signaling pathways targeted by these drugs play a critical role in the formation, maturation and survival of oocytes and follicles. Indeed, the c-Kit, a RTK, and its Kit ligand (Kit-L) are notably important for establishment of primordial germ cells, primordial follicle activation, oocytes survival and growth and granulosa cell proliferation [13]. Platelet-derived growth factor receptor (PDGFR) and its ligand also play a role in primordial follicle activation and could be involved in the angiogenesis during corpus luteum formation [14]. In addition, the angiogenic vascular endothelial growth factor (VEGF) is largely expressed by oocytes and somatic (granulosa or theca) cells. The VEGF/VEGF-receptor (VEGFR) system also plays a role in follicle selection [15] and in luteinisation/maintaining of corpus luteum function [16].

Sunitinib is an oral multikinase inhibitor, and an effective antiangiogenic and antitumoral drug approved by the U.S. Food and Drug Administration and the European Medicines Agency for renal cell carcinomas, pancreatic neuroendocrine tumors, and gastrointestinal stromal tumors [17,18]. It is currently developed for pediatric oncology indications notably the refractory solid tumors and acute myeloid leukemia [19–21]. Sunitinib multitargets VEGFR, PDGFR and c-KIT signalling pathways [17], so we hypothesize that this compound could exert a negative impact on ovarian function, which could have severe consequences in terms of fertility for girls and young women. Its toxicity on the ovarian reserve is questionable. Anti Müllerian hormone (AMH), a TGFβ family member, is expressed in granulosa cells of preantral and small antral follicles and is considered today as a useful marker of the number of growing follicles [22]. This hormone exerting its action through the AMH receptor2, is considered as a good indicator of follicular ovarian reserve in mammals [22]. This marker is currently measured to evaluate gonado toxicity after cancer therapy.

The present study was designed to evaluate the preclinical impact of sunitinib on ovarian function using mice as an animal model treated by oral administration of the drug.

## Materials and Methods

### Compounds

Sunitinib or SU11248, (N-[2-(diethylamino)ethyl]-5-[(Z)-(5-fluoro-1,2-dihydro-2-oxo-3H-indol-3-ylidine) methyl]-2,4-dimethyl-1H-pyrrole-3-carboxamide) was from LC laboratories (Woburn, MA). For *in vitro* experiments, sunitinib stock solutions were prepared in 100% dimethylsulfoxide (DMSO). Equivalent DMSO concentration (0.1%) served as vehicle control. For *in vivo* experiments, sunitinib was dissolved in 80mM citrate-buffered (pH 2.5) solution for a final concentration of 10 mg/mL.

### Mice and treatment

**Fig 1:**
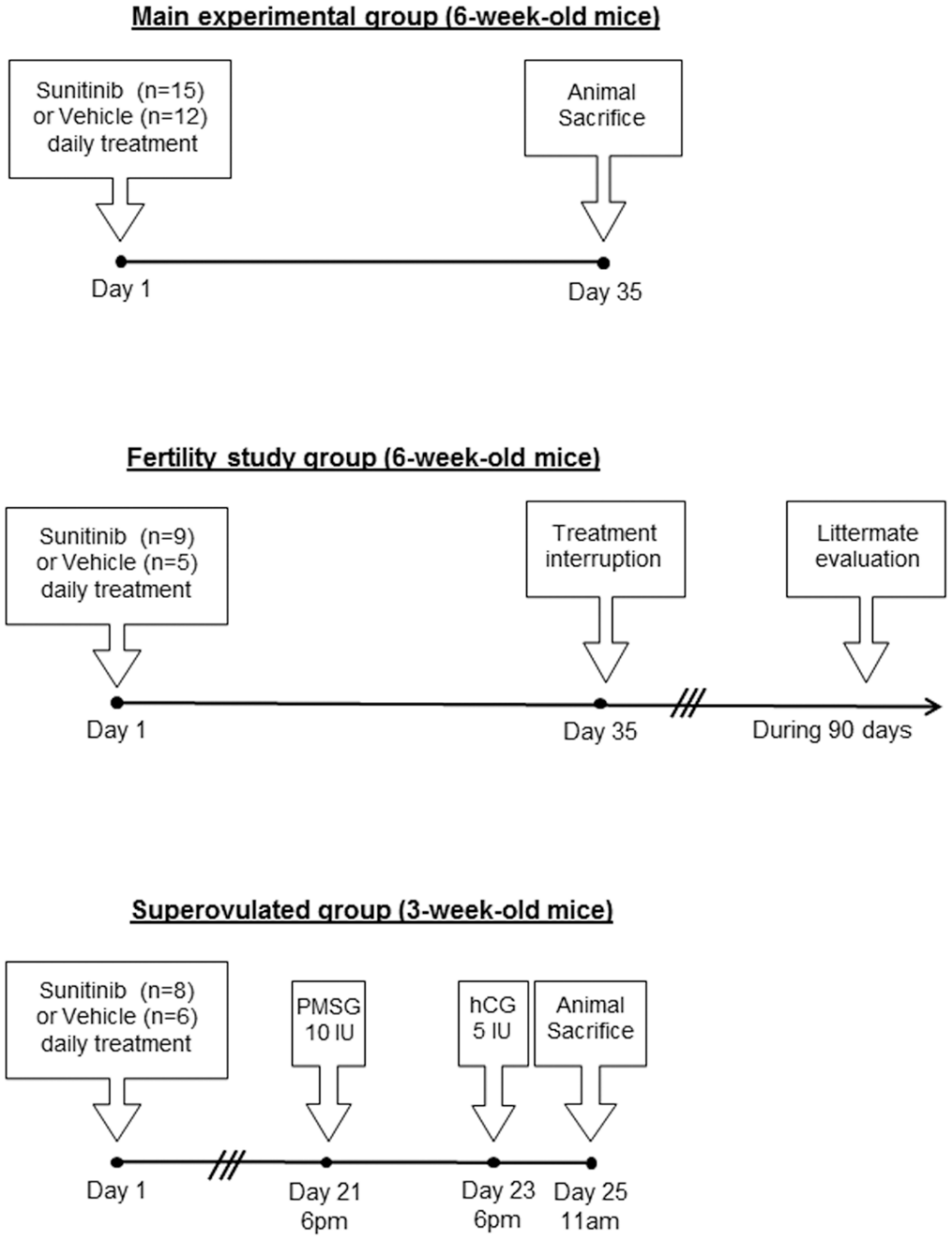
Study design. Six-week-old female mice received [*per os*, once daily (5d/wk)] either sunitinib (n = 19) or vehicle (n = 12) during 5 weeks, and were sacrificed at day 35. Another group of 5-weeks-sunitinib (n = 9) or vehicle-treated (n = 5) mice were mated immediately after treatment withdrawal at Day 35 in order to study their fertility. A third group of 3-week-old female mice received sunitinib (n = 8) or vehicle (n = 6) during three weeks, and then were superovulated by intraperitoneal injection of 10 IU pregnant mare’s serum gonadotropin (PMSG) at 6pm, followed by intraperitoneal injection of 5 IU human Chorionic Gonadotropin (hCG) 48 h later. Animals were killed 17 h after the hCG injection.

Six-week-old female mice (purchased by Janvier, France) were provided ad libitum access to food and water (deionized) and were housed under conditions of constant temperature (21°C ± 2°C), humidity (min. 50%), and lighting (12L: 12D, lights-on at 0700 h). They were randomized into 2 groups: mice then received [*per os*, once daily (5d/wk)] either vehicle (citrate-buffered, pH 2.5) or sunitinib (50 mg/kg/d) during 5 weeks as described in the literature [23]. Body weights were monitored weekly. Mice were killed after an intraperitoneal anesthesia (a ketamine/xylazine mix), tissues removed and stored for subsequent gene expression and histological analyses. Another group of control and treated mice was kept for a fertility trial, which was performed during 3 months after treatment withdrawal. The treatment protocol and the time frame of the experiment are presented in Fig 1. The animal facility was granted approval (N°C94-043-12), given by the French Administration (Ministère de l’Agriculture). All procedures were approved by the local ethic committee Consortium des Animaleries Paris Sud (CAPSud) (N°2012–021).

### Analysis of estrous cyclicity

Estrous cycles were monitored daily by vaginal smears taken at the same time over 5 weeks and analyzed for the predominance of either lymphocytes, or nucleated epithelial cells or keratinocytes [24]. One drop of PBS from a Pasteur pipette was expelled into the vagina, aspired, and then transferred to a microscope slide. The duration of each cycle stage was also determined by calculating the percent of days in each stage during a period of 35 days.

### Ovarian histology

For histological examination of ovarian morphology, ovaries were dissected and fixed for 5 hours in 4% PFA, washed, paraffin-embedded, and sectioned. After routine histological procedures, 8-μm sections were mounted on glass slides and stained with hematoxylin and eosin. Follicle count was performed as described previously [25]. In brief, based on the mean diameter of the follicle, growing follicles were divided into four classes: small preantral (20–170 μm), large preantral (171–220 μm), small antral (221–310 μm), and large antral (>311 μm). Growing follicles were counted in every fifth section. Primordial follicles were counted in every second section. In addition, the presence of recent corpora lutea was determined.

### Superovulation test

Three-week-sunitinib treated and control females were superovulated by intraperitoneal injection of 10 IU pregnant mare’s serum gonadotropin (PMSG; Sigma-Aldrich, Saint-Quentin Fallavier, France) at 6pm, followed by intraperitoneal injection of 5 IU human Chorionic Gonadotropin (hCG; Schering-Plough, Courbevoie, France) 48 h later. Animals were killed 17 h after the hCG injection. Oocytes were extracted from the ampulla and counted after enzymatic dissociation from the surrounding cumulus with hyaluronidase (Sigma; 100 μl type IV-S; 10 mg/ml).

### Amh gene expression analysis

Total RNA was extracted from ovaries using TRIreagent solution (Ambion, Saint-Aubin, France). Quantitative real-time PCR was performed as described previously [26]. After DNAse I treatment (Invitrogen), RNA was reverse transcribed and used for quantitative RTPCR (qRT-PCR) using the Power SYBR Green PCR Master Mix (Applied Biosystems). Final primer concentrations were 300 nM for each primer. Reaction parameters were carried out on a StepOne Real-Time PCR System (Applied Biosystems). Reaction parameters were as follows: 95°C for 20 seconds, then 40 cycles at 95°C for 1 second and 60°C for 15 seconds. Controls without reverse transcriptase and without template were included to verify that fluorescence was not overestimated by residual genomic DNA amplification or from primer dimer formation. Moreover RT-PCR products were analyzed in a post-amplification melting curve to ensure that a single amplicon was obtained. Quantification was performed by the standard curve method. For preparation of standards, amplicons were purified from agarose gel and subcloned into a pGEM-T Easy plasmid (Promega), then sequenced to verify the identity of each fragment. Standard curves were generated by serial dilutions, spanning 6 orders of magnitude, yielding a correlation coefficient of at least 0.98 in all experiments. For all experiments, PCR efficiency was close to 1, indicating a doubling of DNA at each PCR cycle, as expected. *Ribosomal 18S* were used as reference gene for data normalization. Relative expression of *Amh* gene is expressed as the ratio of attomoles of this specific gene to femtomoles of *rRNA* reference gene. Standard and sample values were determined in triplicate from three independent experiments. Results are mean ± SEM and represent the relative expression compared with that obtained with controls. Accession numbers for *Amh* and *18S*, size of amplicons and sense and antisense primers are indicated in S1 Table.

### Hormone measurement

Serum AMH levels were measured with an in house AMH ELISA assay, as described previously [25,27]. In brief, human AMH standards and diluted mouse serum samples were added in duplicate to microplates coated with an AMH-detecting antibody (F2B/7A). Following incubation and washing, the plates were incubated with a biotinylated AMH-capture antibody (F2B/12H), followed by incubation with polyhorseradish peroxidase conjugate (Mast Group Ltd., Merseyside, UK). Next, tetramethylbenzidine substrate (Insight Biotechnology International, Wembley,UK) was added for the chromogenic reaction, which was stopped with 6% (vol/vol) phosphoric acid and the absorbance were read using a microplate reader (Bio-Rad, Hemel Hempstead, UK).

### Cell culture

KGN, a human granulosa cell line [28] and GRAL, a mouse granulosa cell line generated in our laboratory were used for cell culture. These cells were cultured in Dulbecco’s Modified Eagle Medium F12 (DMEM-F12, PAA laboratories, Austria) supplemented with 10% fetal bovine serum (FBS, Biowest, France), 2 mmol/L L-glutamine (PAA laboratories, Austria), 100 units/mL penicillin-streptomycin (PAA laboratories, Austria).

### Western blot analysis

KGN and GRAL cells were treated with vehicle or 1μM sunitinib over 4 hours and then stimulated with 75ng/mL of PDGF-BB during 5, 10 or 30 min. Briefly, cells were lysed in a lysis buffer (150 mM Tris-HCl pH 7.5, 150 mM NaCl, 5mM EDTA, 30 mM sodium pyrophosphate, 50 mM NaF) containing 1% (v/v) Triton X-100, 1% (v/v) protease inhibitor cocktail (Sigma-Aldrich) and 1mmol/L Na orthovanadate. Lysates were clarified by centrifugation at 12,000 x g for 20 minutes. Then proteins (50 μg/lane) were separated by 10% SDS-PAGE and subjected to immunoblots onto nitrocellulose membrane (LI-COR, Lincoln, NE, USA). Blots were incubated for 1h at room temperature in a blocking buffer (LI-COR) before an overnight incubation at 4°C with primary antibodies. Rabbit polyclonal antibodies directed against anti-p42/44 MAPK (9102; 1:2000 diluted), phospho-p42/44 MAPK (9106; 1:1500 diluted) were purchased from Cell Signaling Technology (Saint-Quentin Yvelines, France). Loading controls are performed using antibody directed against tubulin (anti α-tubulin Sigma Aldrich). After washes, blots were incubated with an IRDye 800-conjugated affinity purified anti-rabbit IgG second antibody (1:10,000; Thermo Scientific, France) for 1 h at room temperature. Proteins were visualized with an Odyssey Fc apparatus (LI-COR).

### Statistical analysis

Data are expressed as means ± SEM and analyzed using a non-parametric Mann Whitney test with use of the computer software Prism 5 (GraphPad Software, San Diego, USA). Statistical significance is indicated at P values <0.05, 0.01 and 0.001.

## Results

### Mice cyclicity

**Fig 2:**
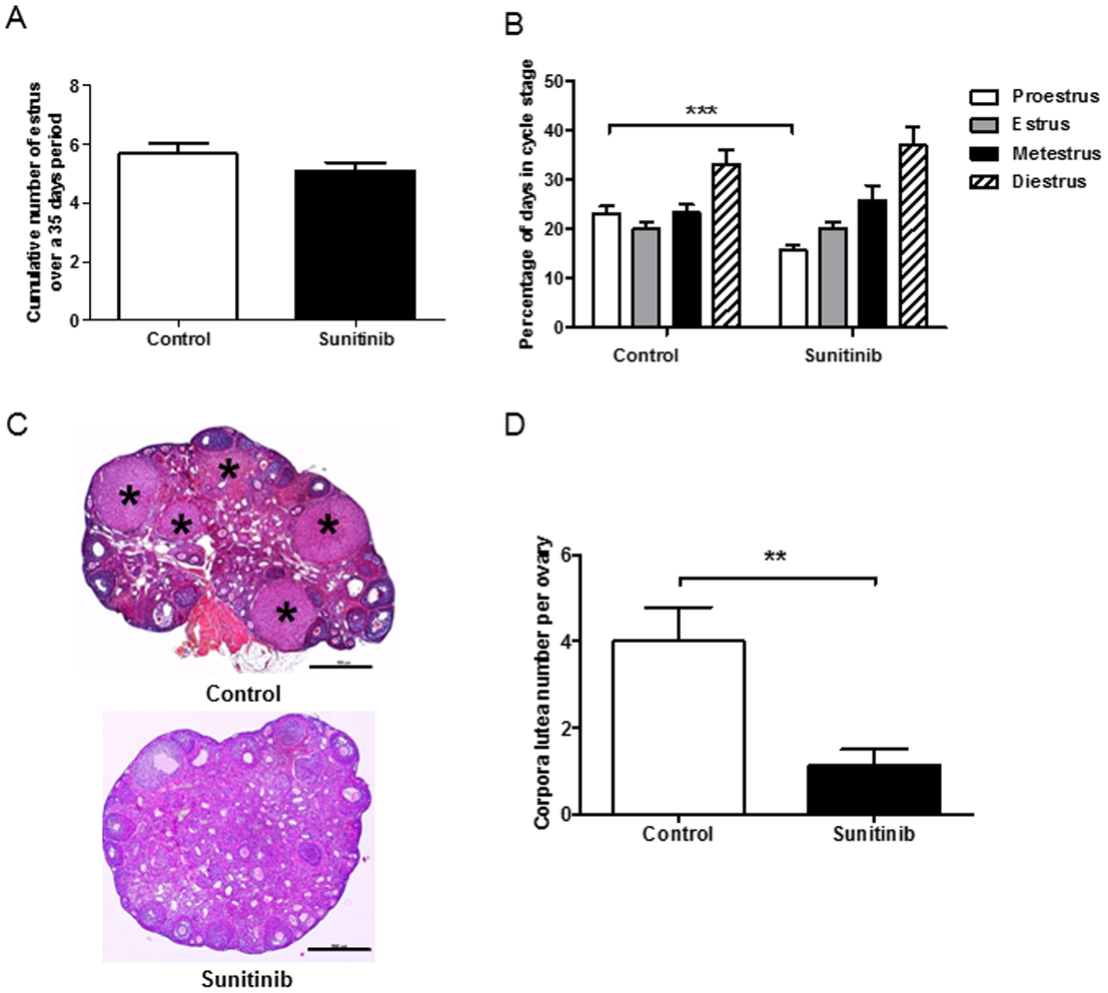
Mouse cyclicity and histological analysis of ovaries. A. Representative mean number of estrus along 35 days in the control group (n = 12) and the sunitinib-treated group (n = 15). B. Representative percentage of days in each cycle stage over a 35 days period in both groups. Each bar represents mean with SEM. C. Representative ovarian histological sections from control mice and sunitinib treated mice. Asterisks indicate corpora lutea (CL), reflecting ovulation rate. Bar scale = 500μm. Note the absence of CL in the ovary of a sunitinib-treated mouse. D. Mean number of corpora lutea per ovary after 35 days of vehicle or sunitinib administration. Quantitative analysis revealed a marked decrease in corpora lutea number in the sunitinib group (n = 15) versus the control group (n = 12). **p < 0.01.

To evaluate the impact of sunitinib on hypothalamo-pituitary-gonadal axis, 6-week-old female mice were treated with sunitinib or vehicle by oral gavage along 35 days. Treatment did not affect the body weight gain profile (data not shown) and no apparent toxicity was observed. Using vaginal smears, an index of cycle stage, we demonstrated that both groups presented with a normal pattern of cyclicity (Fig 2A). There was no apparent difference between the two groups in the cumulative estrus number along 35 days. The analysis of the percentage of days in four different stages (Fig 2B) revealed a percentage of days in proestrus significantly lower in sunitinib treated mice, but a similar percentage of days in estrus stage.

### Ovarian morphology

**Fig 3:**
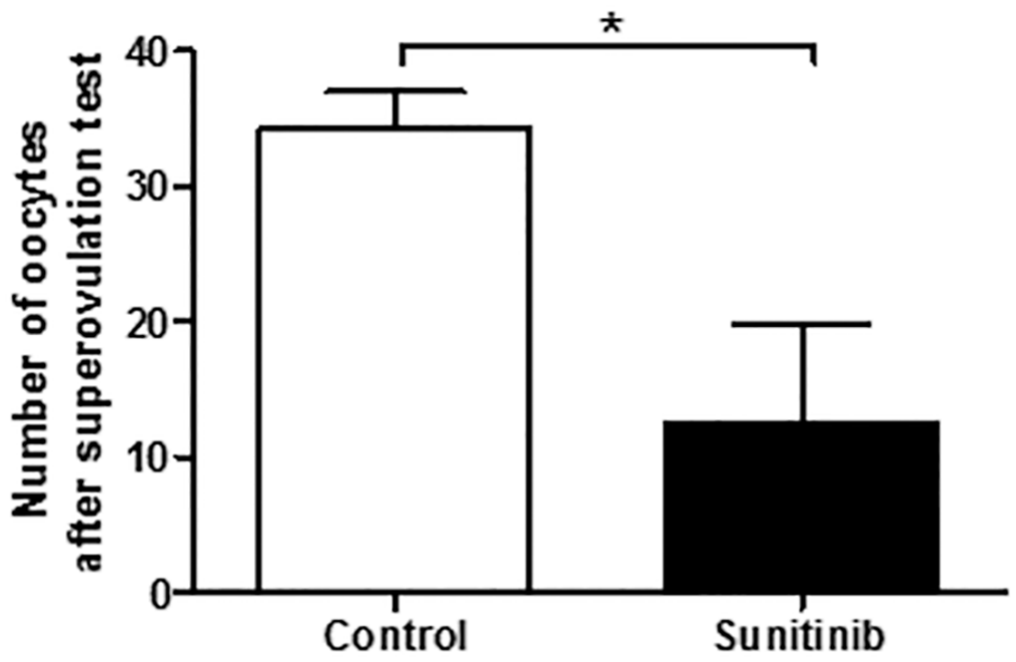
Superovulation test. Number of oocytes obtained after superovulation test in sunitinib-treated (n = 8) and control mice (n = 6).

Ovarian histological analysis revealed that despite the presence of follicles at all stages of maturation in both groups, ovaries of sunitinib-exposed mice exhibited a drastic decreased number of corpora lutea per ovary (1.1 ± 0.38) as compared to those of control mice (4 ± 0.79, p<0.01) (Fig 2C and 2D). This finding suggests an effect of sunitinib treatment on ovulation rate and/or the subsequently luteinization process required to maintain pregnancy in mouse. To investigate these hypotheses, mice were submitted to a superovulation test. We found a significant decrease in the number of oocytes ovulated in sunitinib-treated mice compared to control mice (p<0.05) (Fig 3). This result strongly argues in favor of a peripheral defect rather than a central pituitary deficiency.

### Ovarian reserve analysis

**Fig 4:**
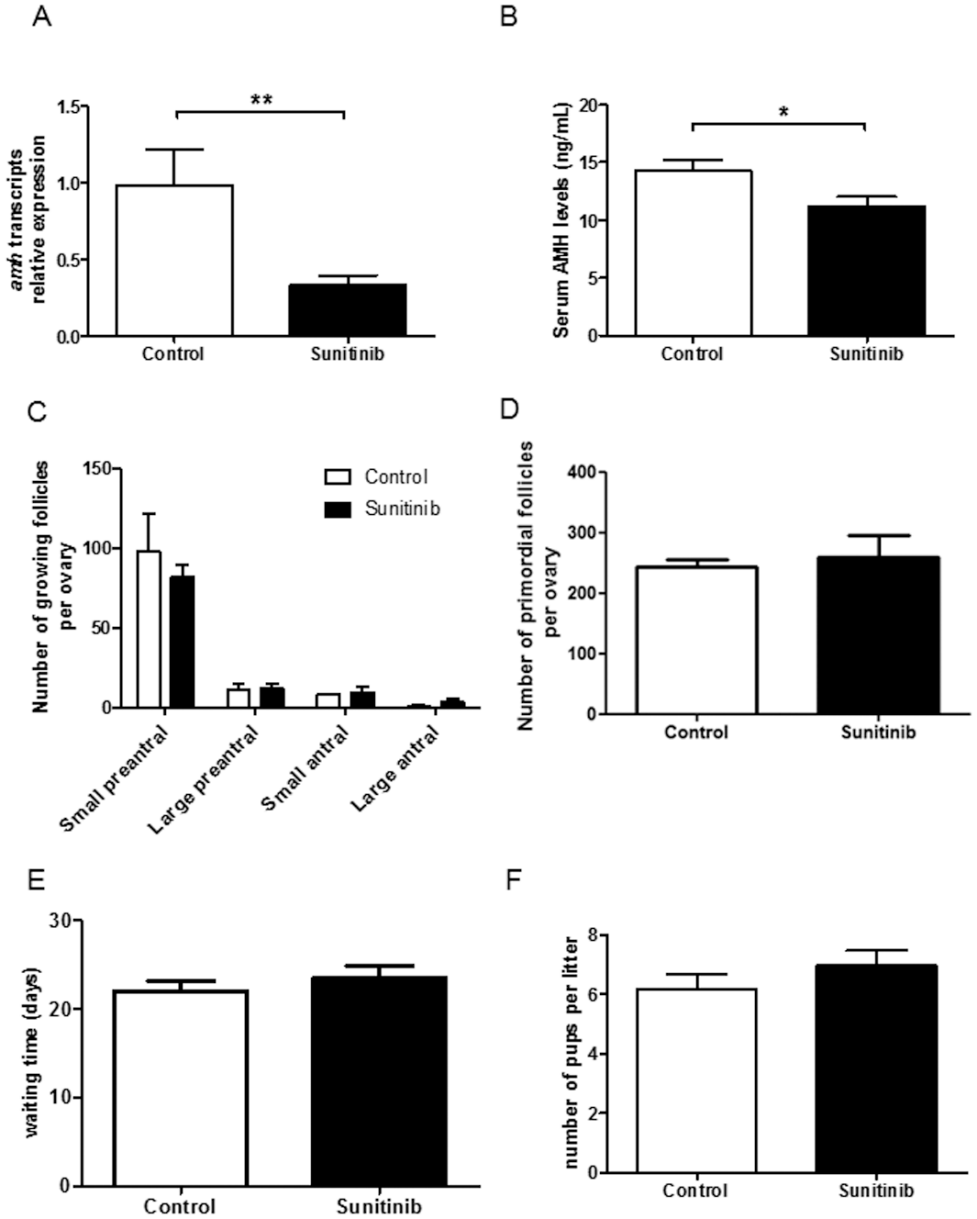
Ovarian reserve evaluation after sunitinib or vehicle treatment. A. Expression of ovarian *Amh* transcripts normalized to *18S* RNA in mice treated by sunitinib (n = 15) compared with control mice (n = 12) **p<0.01. B. Serum AMH levels in sunitinib-treated mice (n = 15) compared with control mice (n = 12). *p<0.05. C. Growing follicle counting in sunitinib-treated mice compared with control mice. D. Primordial follicle counting in sunitinib-treated mice compared with control mice. E. Mean delay to obtain first litter in sunitinib (n = 9) and control (n = 6) mice. F. Mean number of pups per litter obtained during 3 months of observation of sunitinib (n = 9) and control (n = 6) mice.

To evaluate sunitinib impact on the ovarian reserve, the values of anti-Müllerian Hormone (AMH) a marker of growing follicles number [25], were measured. The expression of *Amh* transcripts was evaluated in the ovaries of both groups of mice at the end of the sunitinib treatment. We found a significant decrease in *Amh* transcript expression in sunitinib-treated mice, compared to control mice (p<0.01) (Fig 4A). Moreover, serum AMH levels revealed a significant decrease of this circulating hormone in sunitinib-treated (12.0 ng/mL ± 1.16) compared to control mice (14.33 ng/mL ± 0.87, p< 0.05) (Fig 4B). These results suggested that growing follicles could have been altered by sunitinib administration. To investigate this hypothesis, we performed an extensive follicle counting after 5 weeks of sunitinib or vehicle administration. We were not able to find any significant statistical difference concerning growing follicles counting (Fig 4C) and primordial follicles reserve (Fig 4D) in both groups suggesting that ovarian reserve was not affected in sunitinib treated group. Indeed, immediately after withdrawal of 5-wks-sunitinib or vehicle treatment, female mice in both groups were able to obtain litters. The delay to obtain first litter (Fig 4E) and the mean litter size during 3 months of observation (Fig 4F) were not different in both groups. No offspring malformation was observed.

### *In vitro* effect of sunitinib on mouse granulosa cell proliferation

**Fig 5:**
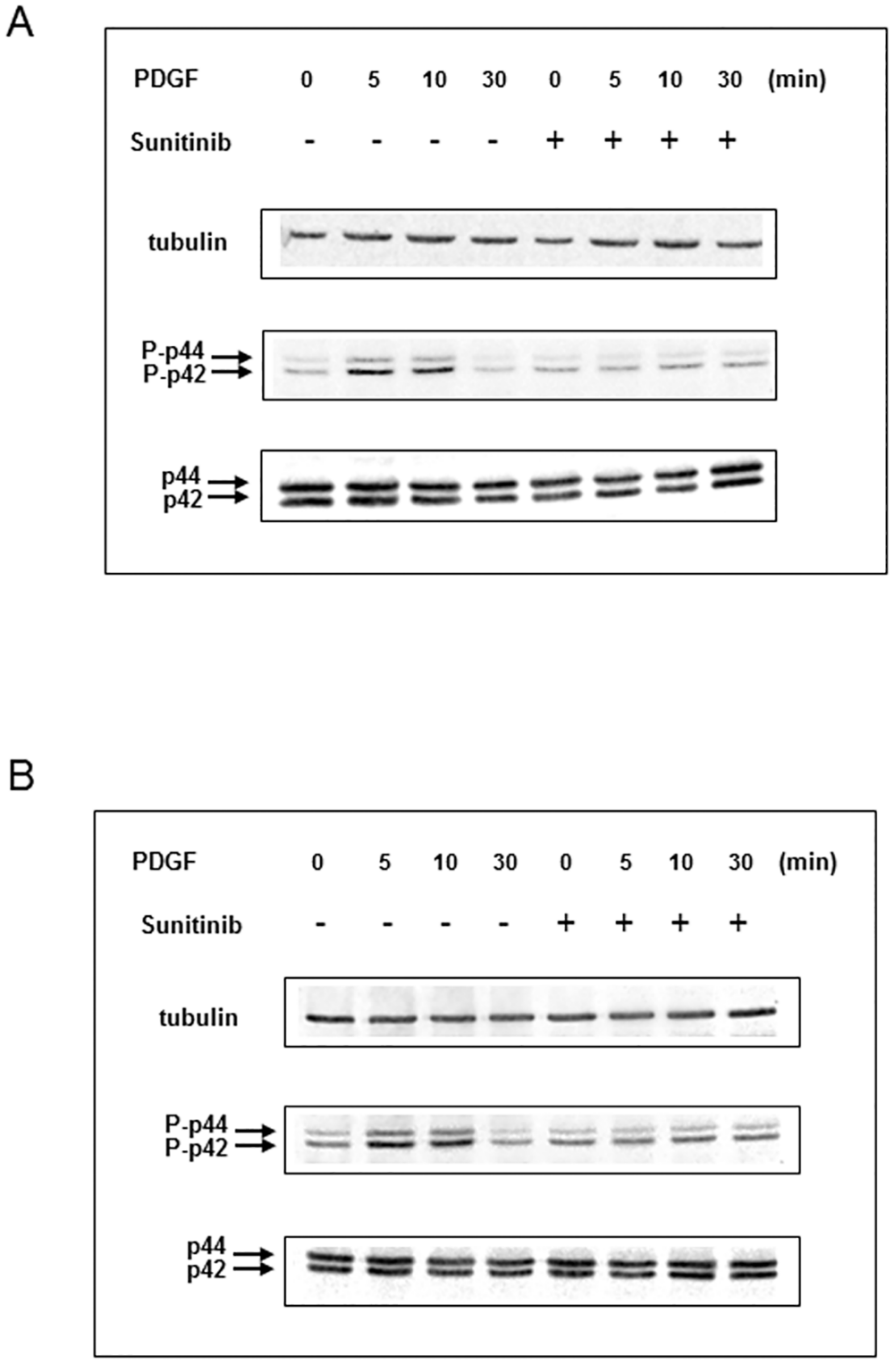
Sunitinib displays an antiproliferative activity. Sunitinib inhibits p42/44 MAPK activation in response to PDGF on human (A) and mouse (B) granulosa cells *in vitro*. Cells were treated by sunitinib or vehicle for 4 hours and subsequently stimulated by PDGF for 5, 10 or 30 minutes (min). Lysates were immunoblotted with anti p42/44 and anti-phospho-p42/44 (P-p42/44) indicated by arrows, as described in the Materials and Methods section. Tubulin was used as a loading control. After PDGF treatment, KGN and GRAL cells demonstrated rapid and transient phosphorylation of p42/44 MAPK reaching maximal level by 10 min and declining thereafter to background levels within 30 minutes. This effect was totally abrogated in presence of sunitinib on both cell lines.

To evaluate the effect of sunitinib on granulosa cells, we tested, as a proliferation index, the *in vitro* effect of sunitinib on p42/44 MAPK phosphorylation by Western blotting experiments on KGN and GRAL cells. The cells were treated by PDGF as a positive control, they demonstrated rapid and transient phosphorylation of p42/44 MAPK reaching maximal level by 10 min and declining thereafter to background levels within 30 minutes. This effect was totally abrogated in presence of sunitinib on both cell lines (Fig 5).

## Discussion

Sunitinib is an oral multitargeted tyrosine kinase inhibitor with potent antiangiogenic activity. It is approved in adulthood to treat renal-cell carcinoma, gastrointestinal stromal tumor [17] and more recently pancreatic neuroendocrine tumor [18]. Over these last years, as other TKIs, this compound has also proved its efficacy to treat solid or hematologic tumors in pediatric oncology and its use is increasing [20,21]. Prolonged administration of TKIs is known to cause endocrine-related side effects such as hypothyroidism and diabetes, but data regarding their effects on gonadal function and subsequent fertility are lacking [29]. Here, we report for the first time in a mouse study that sunitinib could have a negative impact on ovarian function.

The main ovarian phenotype observed in sunitinib-treated mice compared to control mice was the drastic decrease in corpora lutea number. This observation could be the consequence of an ovulation defect and/or a lutenization process inhibition. Otherwise, the inhibition of p42/44 activation, observed in both human and mouse granulosa cell lines after sunitinib exposure, is in accordance to a failure of ovulation process. Indeed, p42/44 activation by LH-surge in human granulosa cells of preovulatory follicles is essential for LH-induced oocyte resumption of meiosis, ovulation and luteinization [30]. The disruption of MAP-kinase pathway in mouse granulosa cells has been demonstrated to be associated with ovulation failure and infertility in mouse [30]. Therefore, sunitinib could mimic in part this phenotype, explaining the drastic decrease of corpora lutea number. This hypothesis was confirmed by the superovulation test showing a significant decrease in ovulated oocytes in sunitinib-treated mice compared to control mice. In addition, VEGF is a mandatory factor required for normal endometrium development [31], and for the formation of corpus luteum, a highly vascularized endocrine organ, which plays a critical role in the maintenance of pregnancy [16]. Sunitinib could have prevented corpora lutea formation. Since animals were able to produce litters in both groups after treatment withdrawal, the sunitinib effects on corpora lutea formation and endometrial function are likely transient in mouse. The reversibility of these latter described effects should be taken with caution because mouse is a polyovulatory species and has a long reproductive life span that could have minimized the phenotype. Reversibility of sunitinib effect on ovulation process should absolutely be evaluated in women.

Regarding the mechanisms of ovarian toxicity induced by sunitinib, it is well known that this drug targets c-Kit and PDGFR which are expressed both in oocytes and granulosa cells respectively. During ovarian development, both c-Kit and PDGFR activation promote the transition from primordial to primary follicle [14]. Thus, we hypothesized that the inhibition of these receptors by sunitinib could impact follicular activation and therefore increase atresia. Nevertheless, no blockade of follicular growth was observed in ovarian histological analysis of sunitinib-treated mice. Follicles at all stages of maturation were observed in both treated and control groups in this study. Additionally, sunitinib exerts an antiangiogenic effect by targeting also VEGFR [17]. Yet it is known that in the ovary, VEGF signaling pathway is required throughout follicular development [32]. Thus, it has already been suggested that intra-ovarian VEGFR inhibition could prevent follicular maturation [32]. Some studies demonstrated that not only apoptotic process but also blood vessel injury can explain ovarian toxicity induced by cancer chemotherapy [33]. As an example, bevacizumab is a monoclonal antibody designed to specifically inhibit VEGF. In a prospective study of 179 premenopausal women randomized to receive chemotherapy with or without bevacizumab, the incidence of ovarian failure was higher (34% versus 2%) in the bevacizumab arm compared to the control arm [34], underlining the fact that specific antiangiogenic effect can lead to an ovarian reserve decline.

To investigate the possible effect of sunitinib on ovarian reserve decline in sunitinib-treated mice, we evaluated AMH levels at the end of the treatment. Serum AMH was significantly decreased in sunitinib-treated mice compared to control mice. We found that the decrease of this circulating hormone in our model could be due to an alteration of *Amh* gene expression by follicles during sunitinib-treatment. Nevertheless, the growing and primordial follicles counting did not reveal any significant decline after sunitinib treatment, demonstrating that ovarian reserve seems not to be affected after 5 weeks of treatment in mice. This suggests a transient toxicity of sunitinib on AMH secretion, which will recover upon time after treatment, without ovarian reserve alteration. This phenomenon has already been described for other anti-cancer drugs [35]. Nevertheless, it remains very important to evaluate in a prospective study the impact of sunitinib on ovarian reserve in girls and young women, by monitoring both antral follicular count and AMH levels before, during and after treatment. In case of follicular depletion induced by the treatment, fertility preservation processes should absolutely be discussed with the patient and/or their parents [36]. In the same line, evaluation of fertility impact of other TKIs should be done in order to improve their toxicity management as well as information given to patients receiving such therapies.

To conclude, our study demonstrates for the first time the negative impact of sunitinib on rodent ovary, especially on ovulation process. This phenotype is reversible and the ovarian reserve does not seem to be affected in mice. Its effect on human ovary should absolutely be evaluated in prospective studies. These new data are important to take into account and could be helpful when fertility preservation methods are discussed before the initiation of cancer treatment in girls and young women.

## Supporting Information

S1 Table Accession numbers and primers used for quantitative PCR. (DOCX)
