# Supplementary material for: The Tyrosine Kinase Inhibitor Sunitinib Affects Ovulation but Not Ovarian Reserve in Mouse: A Preclinical Study
Source: PLoS One. 2016 Apr 1;11(4):e0152872. doi: 10.1371/journal.pone.0152872 (PMC4818017; doi:10.1371/journal.pone.0152872)
Supplement: S1 Table — (DOCX) [file pone.0152872.s001.docx]

**S1 Table. Accession numbers and primers used for quantitative PCR**

| Gene | Transcript accession No | Primers | Temperature | PCR production size |
| --- | --- | --- | --- | --- |
| *Amh* | NM_007445 | F: AGTTCCAAGAGCCTCCACCT  R: ACGGTTAGCACCAAATAGCG | 60°C | 160bp |
| *18S* | NR_003278 | F: CCCTGCCCTTTGTACACACC  R: CGATCCGAGGGCCTCACTA | 60°C | 66bp |
